# Surviving through the kindness of strangers: can there be “wellbeing” among undocumented refugee children?

**DOI:** 10.1080/17482631.2020.1724757

**Published:** 2020-12-09

**Authors:** Åsa Wahlström Smith

**Affiliations:** Department of Education, Communication and Learning, University of Gothenburg, Gothenburg, Sweden

**Keywords:** Undocumented refugee children, deportability, wellbeing, resilience, anthropology

## Abstract

**Purpose**: The paper exames notions of health and wellbeing in the context of radically retracted rights to political asylum. It questions the tendency in previous research to regard the political economy of refugee protection as a parallel issue to a range of factors affecting children’s health.

**Methods**: Based on ethnographic research with 19 undocumented refugee children in Sweden, the paper illustrates ways in which the deportation regime conditions participants’ health.

**Results**: Findings show that children lived with precarious status for the better part of their childhoods, alternating between undocumented and asylum seeking statuses. Participants accessed formal rights to education and health through complete or relative strangers at risk of exposure to authorities. The paper argues that conceptualisations of refugee children’s suffering in terms of risk and protective factors are redundant in this context. Moreover, deportability, protracted refugee situations and deprived material conditions are not unique to undocumented refugees, but characterise most refugee children’s lives in welfare states today.

**Conclusion**: In relation to the plight of the refugee child, wellbeing seems to refer to an abstract ontology of desirable states of the human experience, far removed from the real day-to-day lives of individuals shaped by social suffering and structural violence.

So you think you can tell

Heaven from hell

Blue skies from pain

Can you tell a green field

From a cold steel rail?

A smile from a veil?

Do you think you can tell?

- Pink Floyd 1975

## Introduction

The lyrics by Pink Floyd help us think about the discord between the description of refugee children’s health and wellbeing in research and their phenomenological experiences. Specifically, the paper examines notions of health and wellbeing in the context of radically retracted rights to political asylum. It suggests that studies concerned with the health and wellbeing of refugee children are often negligent of the extent to which the deportation regime (Peutz & De Genova, 2010)—and not the refugee protection regime—has become the defining feature of refugee children’s real life circumstances. Drawing on anthropological research with undocumented refugee children in Sweden, the paper highlights how “deportability” conditioned every aspect of children’s health (Castañeda et al., 2015; De Genova, 2002; Willen, 2012). Children and their families coped and survived with the help of complete or relative strangers under exploitative and precarious conditions. Some of these contacts provided trust, kindness and goodwill which were otherwise rare in their undocumented situations. The argument is that deportability, protracted refugee situations and deprived material conditions are by no means unique to undocumented refugees, but characterize the lives of the majority of refugee children in welfare states today (Crawley, Hemmings & Price 2011).

I begin by positioning my argument in relation to previous research on the health and wellbeing of refugee children. This is followed by a description of the political context, ethnographic research setting and conceptual framework. Here, research participants’ health and wellbeing are conceptualized in relation to children’s rights—which are largely unfulfilled in the present case—and as embodied fear of deportation which conditions all aspects of health (Willen, 2007). After a brief presentation of methodological and ethical issues, I present the ethnographic material illustrating that there is no inherent link between outward signs of risk and protective factors and children’s phenomenological meanings and lived experiences.

## Previous research on refugee children’s health and wellbeing

Though there is no common agreement of how the health and wellbeing of refugee children should be studied and conceptualized across disciplines, policy and practice, there is a growing consensus over its multidimensional aspects (McCarthy & Marks, 2010). As such the concept of wellbeing provides a wide-ranging understanding of health across physical, psychological and social spheres—and more recently, rights, social exclusion and poverty (Chase, 2013; Broad and Robins 2005). Yet there is no consensus on what its core determinants are (Chase, 2013). Psychiatric and psychosocial approaches to children’s health focus particularly on the effects of potential traumatizing experiences during the perceived forced migration cycle (e.g., pre-flight, flight and settlement) (Ehntholt & Yule, 2006; Fazel & Stein, 2002). Children who seek asylum, those granted temporary leave to remain and those with full refugee status have received more attention in research than children in detention and those who have been returned (Bergnehr, 2018; Ehntholt & Yule, 2006; Fazel & Stein, 2002; Kronick, Rousseau, & Cleveland, 2011; McCarthy & Marks, 2010; McFarlane, Kaplan, & Lawrence, 2011; Zevulun, et al., 2018). Recent policy and human rights research has increasingly highlighted the absence of the presumed permanent and durable end stage of the forced migration cycle as well as changing legal definitions of protection (Bhabha, 2009; Crawley, 2006; Lundberg & Lind, 2017; Meloni et al., 2014).

A substantial body of literature evidences depression, anxiety and post-traumatic stress disorder as the most common vulnerabilities in mental health among different groups of refugee children (Ehntholt & Yule, 2006; Fazel & Stein, 2002; McCarthy & Marks, 2010; Vervliet et al., 2014). Studies have furthermore examined refugee children’s health and wellbeing in terms of their strengths, coping, agency and resiliency, rather than vulnerability and disorder (Bergnehr, 2018; Kohli & Mather, 2003; Watters, 2014). Resilience “indicates the possession of several skills, in various degrees, that help the person cope” and achieve positive outcomes in school and social relationships despite challenging or threatening circumstances (Zolkoski & Bullock, 2012, p. 2296). Children are said to be resilient when they are seen to be coping successfully with traumatic experiences, and lead more successful lives than expected despite being at greater risk than average for serious problems (Zolkoski & Bullock, 2012). In a recent example in the present journal, Bergnehr (2018) uses the concept of agency to analyse ways in which children influence wellbeing and acculturative stress in refugee families.

Children’s mental and psychosocial health is usually perceived as debilitated or strengthened by a range of risk and protective factors. Past traumatic events, post-traumatic stress disorder and physical health problems, environmental factors such as poverty, time taken for immigration decisions, isolation and instability are generally referred to as risk factors (Ehntholt & Yule, 2006; Fazel & Stein, 2002). A stable settlement, social support in the host country, and regenerating “a lost sense of belonging and of being in charge of their lives”, have long been regarded as key to help children recover and settle after arrival (Fazel, Reed, Panter-Brick, & Stein, 2012; Kohli & Mather, 2003). Schools are considered to be a particularly important protective factor by facilitating learning, the development of peer- and community relationships, as well as a sense of identity (Fazel & Stein, 2002).

Such blanket assertions must be critically examined in relation to recent retraction of rights to political asylum. Deterrence policies in welfare states across the world include confinement in detention centres, the implementation of more stringent refugee determination procedures and temporary forms of protection (Silove, Steel, & Watters, 2000). Despite support for refugee children at the rhetorical level, the welfare state is increasingly used as a tool for controlling immigration, such as decreasing the costs of supporting asylum seekers (Crawley, 2006). The focus on risk and protective factors of certain groups of refugee children risk understating these political developments. Though the aim is quite the opposite, research on refugee children’s wellbeing inadvertently reproduce an image of the potential of the refugee protection regime to alleviate refugee children’s suffering. A social determinants approach is more fitting in accounting for ways in which immigrants’ experiences and related policies undermine conditions necessary for health, create health inequalities and mortality (Castañeda et al., 2015, p. 377; Marmot, 2012). In the case of refugee children whose rights to asylum have been radically undermined in the past three decades, the political economy of the “deportation regime” fundamentally undercuts health and wellbeing. These issues are further explored in the contextual and conceptual framework below.

## Understanding the health of precarious status children

In health anthropology, conditions of deportability are increasingly analysed as “embodied” fear, such as in the case of severe withdrawal symptoms among asylum seeking children (Eastmond & Ascher, 2011). Embodied fear can be analysed as the ultimate power of the sovereign state over non-citizens manifested in children’s bodies (Eastmond & Ascher, 2011). In this paper, health and wellbeing are conceptualized in terms of the realization of children’s rights on the one hand, and the embodied, phenomenological experiences of deportability on the other (Bradshaw, Hoelscher, & Richardson, 2007; Willen, 2007).

The children in this study were rejected asylum seekers who resided unlawfully in Sweden and were hiding away from authorities to avoid deportation. They had arrived as dependents on their parents’ claims for political asylum. When the parents’ asylum claims were refused and the right to appeal exhausted, the migration authorities had declared the stay illegal under the EU returns directive (EU Dir, 2008/115). In view of the increasing criminalization of “illegal migrants” in the EU, it should be noted that, juridically and ethically, an act can be legal or illegal but not the person. Moreover, residing in the EU on an undocumented basis is not a criminal offence, but an infraction of administrative regulations (EC, 2006, Resolution 1509). Nonetheless, undocumented refugees’ everyday lives are shaped by “deportability”—the constant threat of deportation, even if not immediate or acute (De Genova, 2002).

The children in the study spent most of their childhood with an unresolved migration status. According to immigration policy at the time, families could lodge a new claim for political asylum after four years of no contact with authorities (Aliens Act, 2005, p. 716). Several of the families lodged new claims for asylum during the course of fieldwork. One family went through three “rounds” of applying for asylum in this way, with intermittent periods of being deported to their country of origin, living in hiding there, and fleeing again to seek asylum in Sweden. This illustrates how formal classifications of different sub-groups of refugee children are unsustainable inclusion criteria in ethnographic and long-term research as the different legal statuses are changing for the individuals involved. It reflects also the presence of a growing category of stateless and “precarious status” children that are not protected by welfare states (Bhabha, 2009; Meloni et al., 2014) and the unlikely positive outcome of asylum claims (Chase, 2013; Watters, 2014). The politics of asylum and policies of deterrence of recent decades has led to a situation in which the deportation regime more accurately describes the statecraft and management of refugees than the intentions of the 1951 Refugee Convention (Gammeltoft-Hansen, 2014; Peutz & De Genova, 2010). In de Genova’s words, with “militarized border policing and the expanding purview of securitization in all aspects of travel and transit”, internment and deportation are exercised to the extent that they have become a global regime (De Genova, 2010, p. 34). The asylum determination process should in this perspective be regarded as an integral part of upholding the violent system of the deportation regime (Lundberg & Lind, 2017). A general culture of mistrust is pervasive in asylum determinations. In the balancing act between migration control on the one hand and children’s rights on the other, assessments of asylum-claims are predisposed to rejecting claims in Sweden and worldwide (Bhabha, 2004; Lundberg, 2011; Lundberg & Lind, 2017).

### The Swedish political context

Swedish law specifies that children who reside unlawfully in the country have rights to education, health- and medical services (Regeringen, 2012a, 2012b).^1^

While this represents a more favourable situation than children have in many other countries, children access these rights at the risk of discovery of their undocumented status. The duality of undocumented children’s legal and social position has been highlighted previously (Lind, 2016; Sigvardsdotter, 2013; Wahlström Smith, 2018). Living off the grid in the Swedish welfare state is particularly difficult and to do. For these reasons undocumented refugee children often “hide in plain sight”, attempting to conceal their migration status in everyday interactions, including to long-term friends (Wahlström Smith, 2018). A civic registration number (*personnummer*) is required by individuals to access welfare services. The registration number is connected to the person’s residency and is updated in a universal computerized system when people change home address. Undocumented persons do not possess this number as they are formally unentitled to welfare services and/or to reside on the territory.^2^ It has also gained social acceptance as an administrative convenience tool to keep track record of individuals in commerce and public services generally, facilitated by the digitalization of services at large. For these reasons, there is almost no aspect of everyday-life that is disconnected to the civic registration number in one way or the other. Public transports, registry at school, library and leisure services, billing and prepaid contracts are hard to access without the civic registration number. In this way, the residency of the person, via the civic registration number, constitutes an important surveillance mechanism of non-citizens (Sigvardsdotter, 2013). Target numbers and police efforts to find and deport rejected asylum seekers have also intensified in recent years. The boarder police have requested information about the whereabouts of undocumented persons from social services, hospitals, and housing associations. Police act on tip-offs from the public as methods to find rejected asylum seekers. A recent infamous example is the arrest and subsequent deportation of undocumented families attending a church summer camp (Lind & Persdotter, 2017).

## Methods

The paper is based on 18 months’ anthropological research and thematic coding and categorizing analysis (Gibbs, 2007; Bernard, 2006). Data collection took place during September 2012- July 2013 and intermittently in 2014. The research includes 29 individuals in total, of which 10 were parents or guardians and 19 were children between 6–17 years of age.^3^ Of the children, there were 11 boys and eight girls. Research participants came from Afghanistan, Iran, Iraq, Lebanon, Bosnia-Herzegovina, Kosovo, and Sri Lanka. Research techniques included participant observation in homes and on outings with children, interviews and informal conversations, as well as task-based and creative research techniques, such as children’s photographs of things that made them feel happy, sad, angry and safe (Wahlström Smith, 2018). There were 53 interviews in total, 46 child interviews, and seven adult interviews. Children and adults self-reported fluency and comfort in spoken Swedish and did not require interpreters, except in the case of three adults in which interpreters assisted via telephone.

The central research question concerned ways in which children’s subjective experiences of deportability conditioned their health. Examples of specific questions to children included the somatic symptoms children had and children’s own explanation of why they came about; the nature of children’s friendships (did they have “best friends” and what could be revealed and not revealed to friends?); what children thought were most difficult to cope with; and what helped them cope.

The risk of detection was central to the study population and gave rise to several methodological and ethical issues. Regarding access, I approached potential study participants through individuals they trusted in voluntary networks. These were individuals, organizations and churches that provided support for undocumented migrants, including medical assistance and referral, food, clothes, housing, and social activities.

Informed consent was treated as a continuous process rather than a one-off event (see Düvell et al., 2010; Hugman, Pittaway, & Bartolomei, 2011). The participating children and parents/guardians were informed about the aim of the research, that their participation was voluntary and could be withdrawn at any time, and that their anonymity was safeguarded. In cases where children were under 15 years of age, I asked for both children’s and parents’/guardians’ informed consent. In other cases, when the child was 15 years of age or older, I asked only for the child’s informed consent.

The study obtained ethical approval by the regional ethical review board in Gothenburg. Since fear of detection and deportability was integral to study participants’ daily lives, it was necessary to go beyond the minimum, universal ethical requirements (Düvell et al., 2010). Participants’ fear and extremely vulnerable position were of central concern in methodology and ethical conduct. Risks involved that authorities would come to know ways in which rules are circumvented, and thereby reducing “the space for life-saving creativity and flexibility in remaining invisible” (Polzer & Hammond, 2008, p. 418). The undocumented situation thus influenced the nature and kind of data that could be gathered and presented at particular points in time (American Anthropological Association [AAA], 1986). I paid special attention to children’s social position of deportability, which was inherently different to adults’, regarding both individual’s own assessment of risk and issues of power relative to the researcher (Punch, 2002; Wahlström Smith, 2018). Building trust and getting to know participants over time was essential for participants to impart their personal experiences. I first conducted background interviews on the family situation with adults to understand the vulnerability they experienced and what might constitute painful memories that should not be broached. I began the research process by asking general questions, and only later, after their consent, to ask for personal and individual accounts. Some identifying details have been altered to avoid identification of study participants. The names are pseudonyms.

## Childhoods “in hiding”—deportability as a determinant of health

In the study several dimensions of deportability as a determinant of health and its embodied manifestations could be discerned. The threat of deportation and the exclusion of the right to remain on the territory gave rise to a destabilized sense of personal safety and protection which affected all other areas of informants’ lives and presented itself as somatic (e.g., embodied) symptoms. Consequently, study participants constantly feared the broken trust of others and thus had difficulty claiming the formal rights that children had (e.g., to education and health). Children experienced high levels of emotional distress symptoms, yet they were also compelled to hide the distress and fear from outsiders and wanted to be seen to “cope” outwardly. They lacked adequate standard of living, which included hunger, poverty, homelessness, and overcrowded housing. I describe these dimensions in turn below.

For most children in the study, becoming undocumented was a dramatic change in their lives. It often meant that they suddenly left the town where the Swedish Migration Agency had placed them during the asylum seeking process, where children had attended school and made friends, to move to larger urban areas in search for voluntary supportive networks. One father described the change from living as an asylum seeker to becoming undocumented in the following way:

“My children went to school and were doing well. We received grants from the Migration Agency. The children played football and were intent on staying here. After the third negative decision, we were called to the Migration Agency. That is when the children got the decision that we would not be able to stay and the children fell apart. The school referred them to a child psychiatrist. We asked the Migration Agency for permission to see the psychiatrist, but they denied the request and said we were to be deported immediately. My children were badly affected. We went to [name of city] to live in hiding. If we were to be returned I would be killed and no one would be able to take care of my children. That is what would happen. It is not for my own sake that I feel fear, but for my children’s future.”

For other children, the change in migration status was less dramatic. Some families already had a network in place, and housing and work in the informal economy. Children in these families experienced less changes during times in-and-out of hiding, often remaining in the same school and neighbourhood as before. Some others, yet, could never find a long-term place to live, and therefore moved houses every couple of months. Parents were often out of work. Common to all these children were their efforts to present an image of normality at school.

Many families lived in poor and high-crime areas which exacerbated their sense of risk in daily life. Many said that they did not go out at night after 7pm and did not let their children out in their neighbourhood to go to leisure activities. Evian, mother of four, described such worries in the following way:

“We cannot send the children out to play. A couple of months ago, for example, there was a lot of fighting and a gang with shotguns that were shooting. The whole area was full of police. Our daughter was at football training and she called her dad. She said, ‘Don’t come now, but they are shooting out there.’ But my husband just ran and got her. He didn’t even tell me, he just ran. They were shooting for several days. It was in the papers. The police could not do anything.”

Children were therefore often confined to spend much of their time in overcrowded homes. Because of lack of money, and a civic registration number, attending sports- and leisure activities was often limited.

Though children had formal rights to education and health, accessing these rights were not straightforward, but came at the risk that service providers or persons of the general public would inform on them. Many families, therefore, asked around in their networks what schools and health services would welcome them before making contact, and avoided some schools and health services in favour of others. The fear of exposure was forever present for research participants. A telling example was how Sam, an 11-year old boy, described his fear of discovery as he attended an Accident and Emergency Unit. He had slipped and hit his head badly at a public swimming bath. Although Sam was aware that he had the right to medical care, he worried about being queried about his civic registration number and that it would have consequences for the family’s residency. As he was rushed to the A and E, Sam was more concerned about this than his injury. To quote:

“It was not the pain, the blood or the fact that they would stitch me up that frightened me most, but that they would ask for my civic registration number. They would understand that we were without permit and they could report us to the police.”

### High emotional distress and high levels of coping

Children typically conducted themselves in ways that would not draw attention to themselves or further burden parents and siblings with their own suffering. They put much effort into being seen to cope well at school and at home. Though they had high levels of emotional distress they put considerable work into hiding this from others, exhibiting high levels of coping and “prosocial” behaviour, despite considerable distress. Therefore, while children reported that they often worried, had trouble sleeping, experienced stomach ache, nausea, headaches, and night mares, they also talked about the importance of self-control, of upholding a “normal image”, and showing a “brave face” to everyone around them. Hiding their problems was integral to hiding their real living conditions and undocumented migration status. It also reflected a general lack of trust that others could help them, and a lack of sense of security. To do well at school, be well liked, to be considerate and helpful, and to assess the potential risk of any situation before engaging in it, were part of their hiding strategies. One woman who helped out in a family described the children’s lack of security and trust in this way:

“They are frightened. They do not want to create any problems, even the smallest thing. One of them got a kick bike in the back of his head in the school yard. He got a concussion, but he did not want to go to the hospital: ‘No, no, no, it is fine’, he said, ‘it is fine’. The same thing happens with a dentist appointment. They do not want to go. There are so many signs that they do not trust anyone, they feel no security. They do not dare to express feelings or needs. All the time I have to ask them: Are you hungry? Are you thirsty?”

Children talked about a sense of responsibility towards people close to them, a feeling that it was necessary to protect their family from deep-seated feelings of fear, frustration and anger. Hannah, a 16-year old girl, described ways in which she was holding back her feelings from her parents in this way:

“I do not show everything to my parents. They will become very sad themselves, which is not fun when you are in this situation. Instead of thinking about oneself all the time, you have to accept the way things are.”

Ali, 17 years-of-age, had very high levels of emotional distress. When I asked about somatic symptoms and if he was often down and sad, he responded:

“If I’m sad? I cry in bed every night. I don’t want to talk about it. No one in the family knows about it.”

Children held back on the somatic symptoms they experienced. If not directly asked about them, symptoms would likely remain unknown. Children reported symptoms related to a general sense of fear, but also that symptoms were induced by sudden fearful events. Douglas, 11 years of age, described how he could get head- and stomach ache if something unexpected happened to him in school, at home or about town. To quote:

“If we are out or something and if we see a police car or police, then I get a head ache because I end up thinking many things. I fear that they will approach us and ask something and realize we are in hiding [*är gömda*]. The worst that can happen is that they will send us back.”

Children could on one hand be said to be highly resilient and “coping”. However, children’s resiliency and coping came at high personal costs of emotional distress. The headache described by Douglas is an example of embodied fear of exposure and deportation (Willen, 2007).

The ways in which children hid their problems could thus be seen not only as a way to hide their undocumented status, but also part of their general “coping” strategies. Even though they had friends, and some “best” and close friends, rarely did they let their friends know that they were “without permit” (*utan tillstånd*). They avoided friends who misbehaved, shoplifted or cheated because they wanted to avoid being caught out and to get into trouble, both in fear of being reported to the police and expose their unlawful migration status. It was also as a long-term precautionary strategy to avoid standing out from the crowd. If they hurt themselves badly at school, they would try not to let it show; they did not “dare” to get angry. Pro-social behaviour, friendships and educational attainment could therefore be said to mask high levels of distress and suffering. Such issues could be seen as signs of resiliency, wellbeing and coping. Yet, on close examination, deportability was its foundational aspects shaping how children presented themselves.

Adults talked about depression, high levels of fear and anxiety in daily life, similar to that described in Castañeda’s (2009) study as “the illegality syndrome”. This is shown to be more prevalent among the undocumented migrant population, than in the population generally (Andersson, Hjern, & Ascher, 2018). Some parents said that they thought, or had thought in the past, that it would be better if they committed suicide because then their children might have a greater chance to receive residence permits. Both children and parents described the presence of hunger, the lack of money and stable housing in daily life. One father of four related the following:

“I cannot help my sons to make their lives stable. I cannot work legally or study and I cannot meet their needs and demands. It feels humiliating. We are tired. I am tired. I sometimes cannot cope with the children. Sometimes I lock them in another room because I can’t cope with them screaming. It is no life. My nerves cannot take anymore. [Drops a bag of grains on the table]. I had to beg this from a 22-year-old today. I ask everyone we know for money, food and credit in shops. Everyone is tired of me and knows I cannot pay back. It is embarrassing. I am ashamed and my children are ashamed too.”

Mehmed, 15 years-of-age, described the presence of hunger in the home in the following way:

“We live in a flat that belongs to another family. Sometimes they come to visit and then they fill the cupboards with food. But before they come, we have nothing and I am often hungry.”

Another example of poverty and lack of money was Douglas and his family who survived on the parents’ work in the informal economy. If there were not enough work to be had they survived on hand-outs of others. Douglas’ mother often told me about constantly denying her children their material needs and of periodical times of hunger. She related to once having a little bit of spare cash and buying a small bottle of soda for her children to share. The family were waiting at a bus stop, when one of the children put the bottle without the top on a bench, and accidently knocked it over. She related how they all cried about it, knowing they could not buy another soda, and also because of how the incident symbolized the poverty they experienced. She told me about how she used to talk to the children about that one day they would be able to stay in Sweden, that she would get a good job, and that they would fantasize about walking around a supermarket with a big shopping cart in which they would put whatever they wanted.

### Surviving through the kindness of strangers

The families in the study had different kinds of networks and contacts that helped them enter and sustain their undocumented livelihoods. However, some also talked about lack of such networks. One father could not house and provide for his children and had left them in the care of a woman he trusted. He and his children had lived outdoors in the woods. After he had left his children in the care of another, he slept rough for periods of time. He talked about his children being hungry in his care. To quote:

“Sometimes we did not have a roof over our head, not food every day and when the children ate lunch at school, the other children stared at them, and my children were embarrassed. That is why I decided to leave them in the care of another person.”

The father now feared that contacting his children could alert the police. He feared that the phone was tapped, or that the children would mention to others that the father had been in touch and that this would somehow get to the attention of the police.

Some persons in the networks were relatives or other persons the families had known before moving to Sweden and were partly the reason why families had decided to move there. Ali, 17 years-of-age and from Iraq, talked about a wide network of relatives and acquaintances in Turkey, Sweden and North America. Through contacts in the network, his father was able to find work in the informal economy. Other families had no previous contacts in Sweden and built these up over time with voluntary networks, churches and voluntary organizations. Others, yet again, had no such networks and found it impossible to hide “underground”. They felt the only option they had was to cooperate with the border police and to be deported.

The networks were not always supportive and could be exploitative. However, the families had little or no power of leverage to break with their connections as they depended on them. Some parents talked about how their contacts had encouraged them to move to Sweden, promising work and business partnership. However, there was often competition within networks, so that a business deal may not materialize or change into an exploitative situation. Some parents talked about landlords overcharging on rent, knowing the family had nowhere else to go, and no possibility to officially complain.

During the ethnographic research with the children and parents I often felt I encountered the most paradoxical conditions imaginable: Living in one of the richest countries in the world, yet frequently meeting and visiting the homes of informants in my own city who lacked adequate standard of living: food, money, clothes, beds, furniture, and children who were hyper-vigilant and always second-guessing whether a situation could somehow lead to detection from authorities. The stories of the past suffering that had led to their flight, their journeys to safer countries, and the daily struggles they faced where heart breaking. With these stories as a backdrop, the many stories of kindness from “helpers”—individuals of informal networks—that research participants related, stood out as a stark contrast to the dire circumstances I observed. Douglas, for example, related the story of the high-end trainers given to him on his 11th birthday. He told me about one helper of the family, Miriam, who visited the family shortly before Douglas’ birthday. She asked him what he wanted for his birthday, and Douglas responded that he wished he could have a pair of Nike Air trainers. His parents were embarrassed by Douglas wish, and said that was not at all what he needed. Miriam suggested that they went with her to town a few a days later, so that Douglas could pick a present he fancied. The parents insisted that she did not buy him an expensive present and before the event, instructed Douglas to not ask for things he did not really need and that they could buy much cheaper. However, on the day they met in town, Miriam insisted that Douglas went with her alone to whichever shop he wanted and that the parents waited outside. Douglas picked a sport shop and in the shop Miriam told him he should pick what he wanted most of all. Douglas picked a pair of Nike air trainers.

In the study I asked children to take photographs of things that made them feel happy, safe, angry and sad. Children talked about school, friends, family, and leisure activities as spaces that made them feel “happy” and “safe”. Safe and happy spaces were ambiguous as described above, since the undocumented migration status permeated every part of their lives. Nevertheless, children talked about the significance of certain spaces and persons that provided a sense of fun, safety and a *suspension* of the ever-present sense of deportability. Regarding things that made him happy, Mehmed, 15 years-of-age, had taken a picture of an old woman who was sitting in the sofa in the family’s flat. He described what the picture signified to him in this way:

“We call her grandma. She makes me happy. She likes us and helps us a lot. When she comes to visit us she leaves bags of food in the kitchen. She calls my mother her daughter, and my mum calls her mum. She is so kind to us.”

One family said that connections they had made through the children’s school and music activity were the most significant for their survival. The mother talked about a time when they came home and saw a police officer in the stairway in their block of flats and that they managed to run away and hide at a teacher’s place. To quote:

“We lived in another flat before which belong to a man who was dealing drugs. One day when we came home, there was a police officer in the stairs. He was walking up the stairs. I pretended I couldn’t open the door. My husband and children were behind me, and I told them in our language: Do not come up, go out. The police was on the phone and said, ‘yes, they are here’. I ran down. My husband took the fire exit with the children. I ran out, there were lots of people in the street. I did not see the children and my husband. I saw the police go out into the crowd and disappear. I called the music teacher and she said, ‘Yes, come to us’. I found my husband and the children and we went there.”

The family talked about the music school and the teachers as an extended family that provided them with the sense that they really mattered as persons with intrinsic value: The music school collected money for rent and clothes, organized demonstrations for the family, urging the Migration Agency to grant the family leave to remain. When the family received a deportation order, the music teachers told the family: Then they can come take all of us. We are a family now. The children in the family talked about the music school as the safest place they could think of. Johanna, 9 years-of-age said:

“At the music school I feel most safe. I have friends there and everyone is so kind. They say everyone belongs there and it does not matter where you come from.”

## Concluding remarks

The present exploration of children’s accounts of survival and coping with life in undocumented situations has shown that precarious migration status conditions all aspects of children’s health. Children and their families coped and survived with the help of complete or relative strangers under exploitative and precarious conditions. At the same time, some such contacts provided trust, kindness and goodwill which were otherwise rare in their undocumented situations. There was no inherent link in their case between assumed protective factors such as schooling and friendships: these were conditioned by the actual on-the-ground experience of deportability. Children put much effort to be *seen to* cope well at school and at home. They did not want to draw attention to themselves at school and attempted to uphold an image that “all was well”. They refused seeking medical care for a concussion sustained at school in case medical personnel would ask for their civic registration number and know they were undocumented. Presumed protective factors are thus conditioned by deportability and imbued with ambiguity.

Research on refugee children’s wellbeing has proliferated at the same time period that there has been a steady backlash against refugee protection (Gammeltoft-Hansen, 2014). In the case of refugee children whose rights to asylum have been undermined radically in the past three decades, it is insufficient, politically and in a phenomenological sense, to use a language of psychological resilience and coping as the reckoning of their suffering. A paradigm shift is necessary.

It is important here to critically examine the units of observation and analysis in research on refugee children’s health and wellbeing. Legal categories of refugee children should not be treated as static in research. Moreover, there is a potential discord between the reckoning of health in terms of mental health, risk, resilience and coping in relation to certain groups of refugee children. Given that only a minority of refugee children in welfare states are provided permanent and stable residency, the deportation regime must be recognized as the fundament upon which health and wellbeing rests. If lack of rights to asylum and refugee protection are treated on par with many others affecting health, those other factors become misconstrued. In that case, wellbeing of refugee children become a hypothetical and normative construct of desirable states of the refugee experience, far removed from their daily realities (Bevan, 2009; Schües & Rehmann-Sutter, 2013). The paper suggests that health and wellbeing are more usefully conceptualized in terms of the realization of children’s rights on the one hand, and the embodied, phenomenological experiences of political economies on the other.

Limitations of the study include a potential selection bias and non-representability of study population. The participating children and adults were those willing and able to talk about their circumstances, and who had access to local and social voluntary networks through which contact was made. Many undocumented refugees cannot avoid deportation or access schooling and basic livelihood. Second, at the time of research, undocumented refugee children could be granted permanent residence status on other grounds than refugee protection (e.g., exceptionally or particularly distressing circumstances). These regulations were removed during the political changes in 2015 after which Sweden adopted the minimum level of EU asylum law and international conventions (see Lind & Persdotter, 2017). This is but one example of the many ways in which the protection rights of refugee children have been devaluated in Swedish policies of deterrence since the study was carried out. A replication of the study is likely to find an increase of irregularized and undocumented children, at the mercy of exploitative networks and a political situation which is becoming normalized by ruling government parties. Future research should be attentive to ways in which policies of deterrence and the deportation regime has encroached on refugee protection. Research should guard against reproducing “the epistemological standpoint of the state” in which policies of deterrence, deportation and deservingness of health care are represented as a reasonable response in the management of forced migration (Peutz & De Genova 2010; Willen, 2012). If this is not given its due recognition, the discord between signs and signifiers, units of observation and their analysis, cannot be understood.

## Notes

1. The right to health is unevenly distributed across categories of migrant children in Sweden. Current debates concern also exclusion of the right to health in relation to children of EU migrants without formal residency in Sweden (see Zillén, 2019).

2. Rejected asylum seekers may not always or immediately be issued with a removal order, but first denied the right to access the welfare system. The right to seek social and medical services in a local council varies regionally across Sweden and has fluctuated in recent years.

3. Nine of the children were between 11 and 17 years of age, and ten of the children 6 to 10 years of age at our first point of contact.
